# Microcontact Imprinted Plasmonic Nanosensors: Powerful Tools in the Detection of *Salmonella paratyphi*

**DOI:** 10.3390/s17061375

**Published:** 2017-06-13

**Authors:** Işık Perçin, Neslihan Idil, Monireh Bakhshpour, Erkut Yılmaz, Bo Mattiasson, Adil Denizli

**Affiliations:** 1 Department of Biology, Hacettepe University, 06800 Ankara, Turkey; ipercin@hacettepe.edu.tr (I.P.); nsurucu@hacettepe.edu.tr (N.I.); 2 Department of Chemistry, Hacettepe University, 06800 Ankara, Turkey; monir.b1985@gmail.com; 3 Department of Biotechnology and Molecular Biology, Aksaray University, 68100 Aksaray, Turkey; yilmazerkut@aksaray.edu.tr; 4 Department of Biotechnology, Lund University, 223 62 Lund, Sweden; bo.mattiasson@biotek.lu.se; 5 CapSenze Biosystems AB, 223 63 Lund, Sweden

**Keywords:** microcontact imprinting, SPR biosensor, N-methacryloyl-l-histidine methyl ester, *Salmonella paratyphi*

## Abstract

Identification of pathogenic microorganisms by traditional methods is slow and cumbersome. Therefore, the focus today is on developing new and quicker analytical methods. In this study, a Surface Plasmon Resonance (SPR) sensor with a microcontact imprinted sensor chip was developed for detecting *Salmonella paratyphi*. For this purpose, the stamps of the target microorganism were prepared and then, microcontact *S. paratyphi*-imprinted SPR chips were prepared with the functional monomer N-methacryloyl-L-histidine methyl ester (MAH). Characterization studies of the SPR chips were carried out with ellipsometry and scanning electron microscopy (SEM). The real-time *Salmonella paratyphi* detection was performed within the range of 2.5 × 10^6^–15 × 10^6^ CFU/mL. Selectivity of the prepared sensors was examined by using competing bacterial strains such as *Escherichia coli*, *Staphylococcus aureus* and *Bacillus subtilis*. The imprinting efficiency of the prepared sensor system was determined by evaluating the responses of the SPR chips prepared with both molecularly imprinted polymers (MIPs) and non-imprinted polymers (NIPs). Real sample experiments were performed with apple juice. The recognition of *Salmonella paratyphi* was achieved using these SPR sensor with a detection limit of 1.4 × 10^6^ CFU/mL. In conclusion, SPR sensor has the potential to serve as an excellent candidate for monitoring *Salmonella paratyphi* in food supplies or contaminated water and clearly makes it possible to develop rapid and appropriate control strategies.

## 1. Introduction

*Salmonella paratyphi* (*S. paratyphi*) is known as one of the major globally distributed pathogenic bacteria and a leading cause of foodborne diseases [1]. These diseases represent a major threat due to their significantly increased incidence throughout the world [2]. Therefore, it is important to be able to detect pathogenic microorganisms in food to provide real-time quality results [3]. Rapid detection of these microorganisms is necessary in order to prevent the occurrence of foodborne diseases and when outburst of the disease appears, to reduce the spreading. Therefore it is important to develop strategies to ensure a safe food supply [4,5]. 

Conventional methods used to detect foodborne pathogenic bacteria are usually and laborious time consuming. Different experimental stages such as cultivation, biochemical identification and serological confirmation are all included in the routine procedures. Biochemical test kits, antibody-based methods, DNA/RNA-based assays and immunological methods have been developed and applied to detect foodborne pathogens [6]. There are a few developments presented nanosensor-based technologies, are exciting and promising developments since these detection tools have useful characteristics, making them suitable for quick assays combined with reliability and sensitivity [7,8,9,10,11].

Optical sensors are the most extensively used sensors for detecting foodborne pathogens. Use of such sensors offers several advantages, including high sensitivity and specificity, accuracy, relatively low cost, rapid response and portability [3]. In recent years, SPR sensors, the most frequently used optical sensors, have attracted particular and great attention for the detection of different target molecules such as prostate specific antigen, bisphenol-A and glucose [3,12,13,14,15]. 

These sensors make it possible to observe and quantify characteristics of biomolecular interactions on the surface of the sensor in real time with the advantage of label free detection [12]. Several assays to improve surface plasmon resonance (SPR)-based sensors for detecting *Salmonella sp.* have been presented in the literature [16,17,18,19,20,21,22]. Most assays were initially based on use of antibodies as recognition elements. In the last few years, molecularly imprinted polymers have been evaluated in connection to detection of microbial cells, and today one can state that they represent an applicable and efficient method in terms of creating specific and selective recognition sites for a target molecule in polymer matrices [23]. Functional and cross-linking monomers are used for co-polymerization together with the template molecule in order to form template-shaped three dimensional cavities [24]. Therefore, sensors relying on molecularly imprinted polymers (MIPs) offer several advantages such as high sensitivity, selectivity and portability, which make these polymers suitable to be applied in many fields [4,25,26]. To our knowledge there are no reports on assays based on whole cell imprinting related to the detection of *Salmonella paratyphi*.

In this study, a microcontact imprinted SPR biosensor for pathogenic bacteria, *S. paratyphi* was developed. Characterization studies were performed using ellipsometry and SEM. The selectivity of the resulting *S. paratyphi-*imprinted biosensors was examined by using other bacterial strains such as *Bacillus subtilis (B. subtilis)*, *Staphylococcus aureus* (*S. aureus*), and *Escherichia coli (E. coli)*. The results obtained when using MIP and non-imprinted polymers (NIP) chips were compared in order to indicate the efficiency of the MIPs. In addition, real sample experiments with the microorganisms present in a real sample were performed with apple juice. 

## 2. Materials and Methods

### 2.1. Materials 

Strains obtained from the Culture Collection Laboratory of the Department of Biology Biotechnology Division, Hacettepe University (Turkey) were *S. paratyphi* ATCC 9150, *E. coli* ATCC 25922*, S. aureus* ATCC 25923*, B. subtilis* ATCC 23857. The following chemicals were obtained from Sigma Chemical Co. (St. Louis, MO, USA): allyl mercaptan, glutaraldehyde (50%, *w/v*), 3-amino-propyltriethoxysilane (APTES), 2-hydroxyethyl methacrylate (HEMA) and ethylene glycol dimethacrylate (EGDMA). Fluka (Buchs, Switzerland) provided α-α’-azoisobutyronitrile (AIBN). The aminoacid-modified acrylate N-methacryloyl L-histidine methyl ester (MAH) was supplied by Research Group Bioreg (Hacettepe University, Ankara, Turkey). All other chemicals were of analytical grade and purchased from Merck A.G. (Darmstadt, Germany). 

### 2.2. Preparation of Bacteria

*S. paratyphi*, *E. coli, S. aureus* and *B. subtilis* strains were used in this study. The bacterial strains were inoculated into Luria-Bertani broth (100 mL in a 250 mL Erlenmeyer flask). After incubation at 37 °C for 18 h with constant shaking at 150 rpm, measurements of the viable counts were performed by serial 10-fold dilutions in sterile 10 mM phosphate buffered saline (PBS) (pH 7.4). Aliquots of each dilution (0.1 mL) were plated onto Tryptic Soy Agar plates in triplicate. The plates were incubated overnight at 37 °C and the colonies on the plate were counted. The concentration of bacteria was calculated in colony forming units per milliliter (CFU/mL). After incubation, one milliliter aliquot of each bacterial culture was centrifuged at 3300 g for 15 min at 4 °C and the culture supernatant was removed. Each bacterial pellet was washed with 1 mL sterile 10 mM PBS buffer (pH 7.4) by resuspending them in the buffer and then spin them down again for three times. The concentrated precipitate was resuspended in 1 mL sterile water. 

### 2.3. Preparation of Microcontact S. paratyphi Imprinted SPR Chips

#### 2.3.1. Preparation and Modification of the Glass Slides

Glass slides (26 × 75 mm) were cleaned for 5 min each in pure ethyl alcohol and then deionized water before treatment for 20 min in acidic Piranha solution (3:1, H_2_SO_4_/ H_2_O_2_, *v/v*). After washing with water the glass slides were dried with nitrogen gas. Chemical modification of the glass slides to introduce amino groups was performed with 3% APTES in toluene (*v/v*) for 2 h. Washing with toluene removed excess APTES before the glass plates were dried with nitrogen gas. The amino groups were derivatized by adding an excess of glutaraldehyde (3% *v/v*) in phosphate buffer (pH 7.4). After 2 h excess glutaraldehyde was removed by washing with phosphate buffer followed by wash with distilled water before being dried with nitrogen gas. Finally, the *S. paratyphi* cells (200 µL of a suspension of 0.5 × 10^8^ CFU/mL) were added dropwise to the glass surface. The plates were left at room temperature over night for immobilization of the cells and subsequent drying. The slides were rinsed with deionized water and dried with nitrogen gas. They were kept at 4 °C in a closed Petri dish until use.

#### 2.3.2. Modification of the SPR Chip Surfaces

SPR chips (GWC Technologies, S. Rosa Rd Madison, WI, USA) have a gold surface which was modified with allyl mercaptan (CH_2_CHCH_2_SH) according to Yılmaz et al. [27]. A solution of allyl mercaptan (3.0 M) was added dropwise to the SPR chips and incubated in a fume hood overnight. Excess allyl mercaptan was removed by washing with ethyl alcohol before the chips were dried in a vacuum oven (200 mmHg, 25 °C).

#### 2.3.3. Microcontact Imprinting of *Salmonella paratyphi* onto the SPR Chips

The first step was to form a pre-polymerization complex between the monomers. MAH and Cu(NO_3_)_2_·2.5H_2_O were mixed in the ratio of (1:1) for 1 h before a stock solution of HEMA (13 µL), EGDMA (40 µL), was mixed with the MAH-Cu(II) complex for 5 min. Then, the initiator AIBN was added into this stock solution. The SPR chip was placed horizontally and the monomer solution with the initiator was placed on the SPR chip surface. Theglass slide with immobilized *S. paratyphi* was brought into contact with the monomer solution on the chip. Polymerization was initiated by UV light (100 W and 365 nm) and lasted for 20 min under nitrogen atmosphere. Then the glass slide with the immobilized cells was removed and the sensor chip was cleaned with 10 mM phosphate buffer (pH 7.4). The chip was also treated with 10 mg/mL lysozyme solution (in PBS buffer, pH 7.4, 10 mM) for 30 min in order to remove any bacterial residues from surface of SPR chips.

### 2.4. Characterization of SPR Chips

Surface characterization of SPR chips were performed by observation using a JEM 1200 EX Scanning Electron Microscope (SEM, JEOL, Tokyo, Japan). The chip surface was cleaned with distilled water and then, dried with nitrogen gas and coated with Au/Pd. Ellipsometry measurements of the SPR chips’ surfaces were performed using an auto-nulling imaging ellipsometer (Nanofilm EP3, Goettingen, Germany). A four-zone auto-nulling procedure integrating over a sample area of approximately 200 µm × 200 µm followed by a fitting algorithm has been carried out in order to analyse the SPR surface thickness. Phase models including air, polymeric film, gold, chromium and SF10 glass was assumed for SPR chips. 

### 2.5. Real Time Salmonella paratyphi Detection

The reflectivity (ΔR) of light was influenced by binding of material to the sensor chip by SPR imager II system (GWC Technologies). *S. paratyphi* detection was carried out from aqueous bacterial suspension prepared in the concentration range of 2.5 × 10^6^–15 × 10^6^ CFU/mL. Ethyl alcohol (50%, *v/v*) and 10 mg/mL lysozyme solution were used in the regeneration step to disrupt the bacterial cell wall. 

The first step involved equilibration of the sensor chip to 10 mM PBS (pH 7.4) for 200 s (flow rate: 150 µL/min). After reaching the stable resonance frequency, aqueous *S. paratyphi* suspensions (5 mL; 150 µL/min flow rate) were applied to the system and changes in reflectivity (ΔR%) were monitored online until the signal became stable (400 s). Ethyl alcohol (50%, *v/v*) and 10 mg/mL lysozyme solution (in pH 7.4, PBS, 100 mM) were applied to the SPR sensor system in order to remove bound bacterial cells from the polymer surface by breaking the interactions between the cell wall and the polymer (350 s). Lastly, reconditioning by washing away residues of the regeneration solution and products form the enzymatic degradation of the cell wall was performed with PBS (pH 7.4) for 200 s to make the system ready for new injections.

Control SPR chips with non-imprinted polymer (NIP) were also prepared according to the same protocol as that the glass slides used for the imprinting were the same chemistry as the glass plates used for production of MIPs, except that the NIP-related glass-plates did not carrying any immobilized *S. paratyphi*. The NIP chip was used to evaluate the non-specific effect of imprinting of the imprinting chemistry on the affinity of bacterial strains for the surface of the sensor chips without any selective cavities. The responses of these chips to the template and competitive bacterial strains were monitored continuously.

### 2.6. Selectivity of the Microcontact-Salmonella paratyphi Imprinted SPR Chip

Selectivity of SPR sensors was determined by their responses to *B. subtilis*, *S. aureus* and *E. coli* strains. *B. subtilis* and *S. aureus* were included due to the fact they have different cell wall structures. *E. coli* was included due preferred because of its similar cell wall structure and bacterial cellular morphology with *S. paratyphi*. The concentrations of each bacterial suspension were kept constant as 15 × 10^6^ CFU/mL. The NIP-chips were applied in the same manner as mentioned above for determining the non-selective binding. 

### 2.7. Real Sample Experiments and Reusability

Experiments were performed with real samples, in this case in apple juice. The juice was diluted 10 times with PBS (pH 7.4) and then, spiked with *S. paratyphi* in a range of concentrations (2.5; 5.0; 7.5; 10.0 × 10^6^ CFU/mL). *S. paratyphi* was detected repeatedly, using equilibration-injection-regeneration cycles for 5 times. The reusability of the system was examined by evaluating the change in reflectivity at repeated assays with the same concentration of *S. paratyphi* suspension (7.5 × 10^6^ CFU/mL).

## 3. Results and Discussion 

### 3.1. Characterization of Salmonella paratyphi-Imprinted SPR Chips

**Figure 1:**
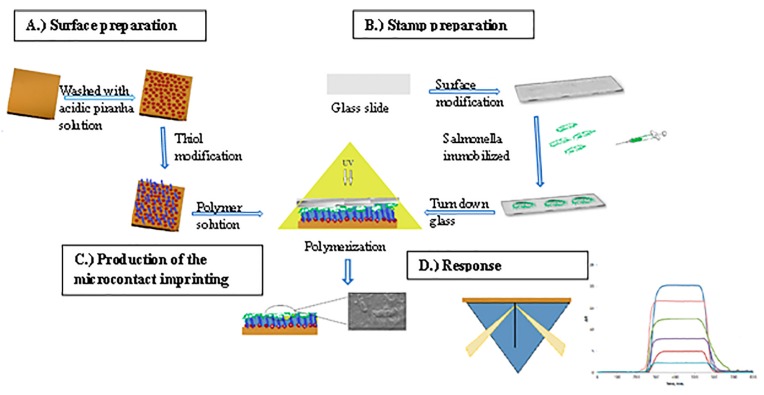
Schematic representation of microcontact imprinting of *S. paratyphi* onto the SPR chip. (**A**) preparation of SPR chip surface, (**B**) preparation of *S. paratyphi* stamps, (**C**) production of the microcontact imprinting and (**D**) response of the SPR sensor system.

*S. paratyphi* imprinted SPR chips were prepared by microcontact imprinting method with the bacterial stamp and monomer mixture covering modified SPR chip surface (Figure 1). MAH (histidine containing specific monomer) was preferred as metal-complexing ligand in order to functionalize the polymer surface and generate specific recognition regions. By this way, selectivity was obtained towards some amino acids present on the cell wall. The interactions between MAH and the cell wall is via glutamic acid residues, diaminopimelic acid. Furthermore, if the cells carry some complex-bound divalent heavy metal ions, then interactions can be expected also there. The interactions between the imprinted cavities made for the target bacterial strain and the polymeric film resulted in high affinity of the bacterial cells for imprinted nano-cavities. It should be stressed that the concept of microcontact imprinting of particulate matter, e.g. bacterial cells, may lead to generation of recognition cavities with two different properties which both contribute to the efficiency in recognizing the target cells. The shape of the cavity is one clear contributing factor to the recognition of the cells and as a second factor, matching chemistry on the surface of the cavity which will selectively interact with the target structures. As is explained in the experimental part, there is a period for the monomers to bind to the surface of the cells before polymerization is initiated. During this period, a sterical arrangement takes place resulting in selective cavities. In connection to the polymerization these conditions are frozen and the MIP structure can after proper treatment start to bind complementary structures (cells of the same character as those used for imprinting). Upon removal of the print structure (the immobilized cells) one is left with a cavity with proper shape of the cells and with a chemistry of the surface of the cavity that matches structures on the cell wall of the target cells.

**Figure 2:**
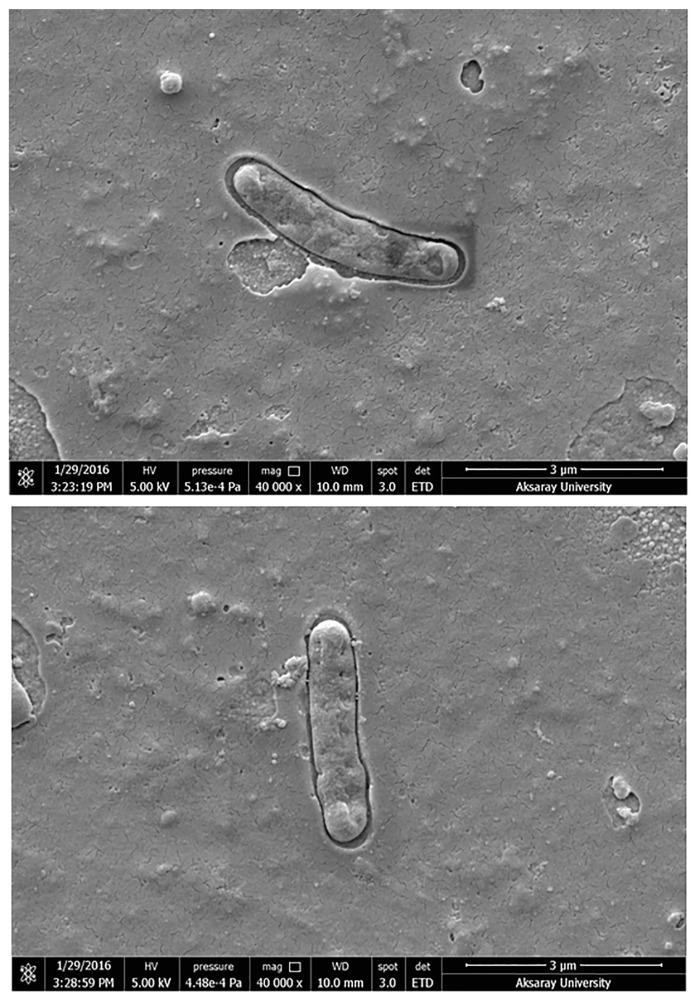
SEM images of microcontact *S. paratyphi* imprinted SPR chip surfaces indicating captured cells.

**Figure 3:**
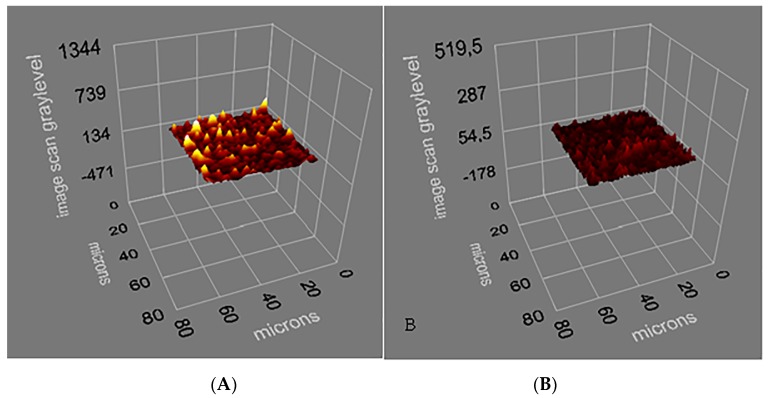
Ellipsometry of (**A**) microcontact *S. paratyphi* imprinted and (**B**) non-imprinted SPR chip surfaces.

Molecularly imprinted polymers (MIPs) represent created artificial receptors and selectively recognition of the target molecule/cell with an efficiency similar to that of natural receptors. Natural receptors have the ability of recognizing the target molecules selectively, but less efficiently recognize by shape of particulate matter. The biological receptors are usually not stable under conditions outside the physiological range [28]. MIPs have great advantages over antibodies due to high stability, low cost and easy preparation [27]. The gold surfaces of SPR sensors were characterized by SEM and ellipsometry measurements. SEM analysis of the SPR sensor chips were performed by JEOL, JEM 1200 EX, (Tokyo, Japan). Bacteria-SEM images indicates the morphology of the *S. paratyphi* imprinted SPR chip surfaces. It is worth noticing that the shape of the cavities are very similar, even though the pictures are taken from different parts of a sensor chip (Figure 2). The thickness of imprinted and non-imprinted polymeric films on the SPR chip surface were determined with ellipsometry measurements as 88.7 ± 1.8 nm and 87.3 ± 0.7 nm, respectively (Figure 3A,B). That the polymer layers were formed on the sensor chips was established by SEM studies and ellipsometry contributed to determine the thickness of these layers.

### 3.2. Real Time Detection of Salmonella paratyphi

**Figure 4:**
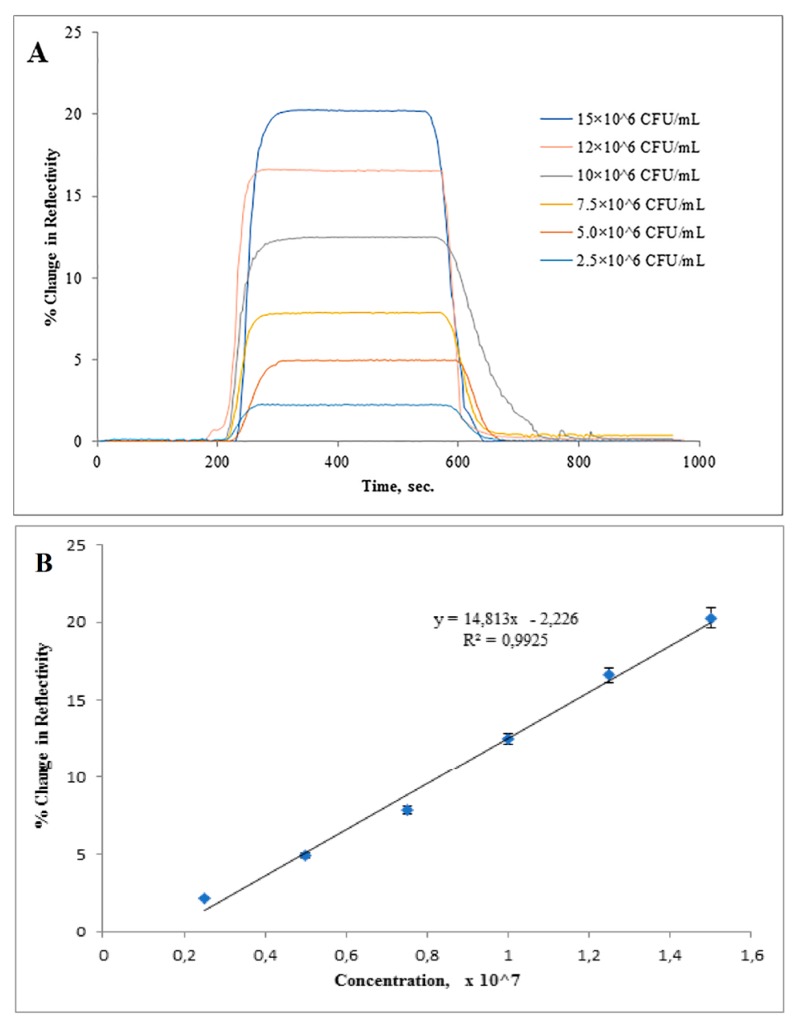
Real time responses of (**A**) microcontact *S. paratyphi* imprinted SPR sensor at different concentrations of *S. paratyphi*, (**B**) calibration curve of *S. paratyphi* obtained at a concentration of (0.25 × 10^7^–1.5 × 10^7^ CFU/mL) under experimental conditions, sample concentration: 1.5 × 10^7^ CFU/mL, flow rate: 200 µL/min, running buffer: PBS buffer, regeneration buffer: 10 mg/mL lysozyme solution, 50% ethyl alcohol.

In Figure 4A, the straight line indicates in the first 200 s indicated baseline. After *S. paratyphi* injection a change in (%ΔR) was monitored as a result of binding of *S. paratyphi* to the recognition cavities and the peak height indicated the amount of material binding of target microorganism. As can be seen from Figure 4B, an increase in response intensity (% resonance frequency shift (%ΔR)) in SPR was registered with the increasing concentrations of *S. paratyphi*.

*S. paratyphi* detection was performed with bacterial suspensions prepared in the concentration range of 2.5 × 10^6^–15 × 10^6^ CFU/mL and *S. paratyphi*-imprinted SPR sensor has a response with a linear relationship to the concentration of cells measured that fits the regression equation *y* = 14.813*x* − 2.226 (*R*^2^ = 0.9925). The limit of detection (LOD) and the limit of quantification (LOQ) were determined to be 1.4 × 10^6^ CFU/mL and 4.5 × 10^6^ CFU/mL, based on IUPAC guidelines. 

In the literature, there are many studies reporting the use of SPR sensors for the detection and quantification of microorganisms in buffer systems and simple matrices. In recent years, they have gained great attention in the fields of health science, drug discovery, diagnosis of infections, environmental and agricultural monitoring [26,29,30,31]. It has been proven that these sensors are also suitable for detecting target molecules/cells in complex media (e.g., blood, urine, stool, food, fruit juice) [32]. 

There are some challenges when using SPR sensors for quantifying cells, due to the size and morphology of the microorganisms. Their huge size leads to slow diffusion to the sensor surface and this could hamper the sensor response and limit detection capability [32].

The pretreatment step during sample preparation has a significant impact on the detection of microorganisms with a SPR sensor. In a previous study, some treatment procedures using cell preparations such as living, heat killed, heat killed and soaked in 70% ethyl alcohol, or detergent- lysed cells were examined and LOD values were found to be respectively decreased. These differences in detection limits can be correlated to the effect of the treatments on size and morphology of the target cell [32,33].

A Biacore system was applied to indicate the detection of heat-killed *Salmonella* strains from groups A, B, D and E, according to Kauffmann-White typing. Antibodies were immobilized on a carboxymethylated Dextran chip by EDC/NHS coupling. A total of 53 different *Salmonella* serovars were detected at 10^7^ CFU/mL. The detection limit in a suspension comprising half *S. enteritidis* and half *S. typhimurium* was demonstrated as 7 × 10^5^ CFU/mL. In two different studies done by the same group, the detection of *S. typhimurium* [17] and *S. paratyphi* [18] was successfully performed. The technique used for *S. typhimurium* detection was similar to what was used when the group previously studied and application for the detection of *E. coli* O157:H7. In terms of *S. paratyphi* detection, the sensor surface was generated by capturing the antibody with self-assembled thiolated protein G. The detection range of these studies was shown as 10^2^–10^7^ CFU/mL. In another study, Koubova et al. used a custom-built SPR sensor for the detection of heat-killed and ethyl alcohol soaked *Salmonella enteritidis* [19]. The SPR chip surface was constructed by immobilizing antibodies raised against these bacterial strains. The LOD value was calculated as 1 × 10^6^ CFU/mL. 

*Salmonella sp.* are a widespread group of bacteria and food products, including pasteurised milk, can be contaminated by these strains. Therefore, it is necessary to detect these microorganisms rapidly since they are one of the main threats to food industry and of course to consumers of the food products. Mazumdar et al. used a cuvette based Plasmonic^®^ SPR device in order to establish a fast and easy immunoassay for detecting *Salmonella typhimurium* in milk [34]. The method was set up as a sandwich model using a polyclonal antibody against the target microorganism. The proposed assay in this study was used to detect *S. typhimurium* in the concentration of 1.25 × 10^5^ cells/mL both in milk and PBS buffer. It can be concluded that there were no differences in detection limits by changing the matrix. Among foodborne pathogens, *Salmonella* serotypes are the causative agents of salmonellosis. In addition to be a main threat to food industry as mentioned above, their detection in water is of great concern in public security. A look at other research reports, polyclonal anti-*Salmonella* antibody was used in order to recognize multiple *Salmonella* serovars simultaneously with the Plasmonic^®^ SPR device. The detection limits were determined to be 2.5 × 10^5^ cells/mL and 2.5 × 10^8^ cells/mL for *S. typhimurium* and *S. enteritidis*, respectively. Milk spiked with both of these bacterial strains was used as a real sample to confirm the assay [20]. 

Our study pointed out that pathogenic microorganisms could be detected without using any antibody and microcontact imprinting has developed into a powerful tool to functionalize surfaces in order to quantify microorganisms. The potential use of microcontact imprinting in combination with SPR sensors provide unique properties. SPR sensors enable label-free, high sensitive and real time detection. These sensors have some additional advantages such as low volume sample requirement and quantitative analysis. When whole cell imprinting was taken into consideration along with the comparison of LOD values reported in literature, the LOD value obtained from SPR biosensor used in our study is among the lowest values in literature.

### 3.3. Selectivity of the Salmonella paratyphi Imprinted SPR Chip

**Figure 5:**
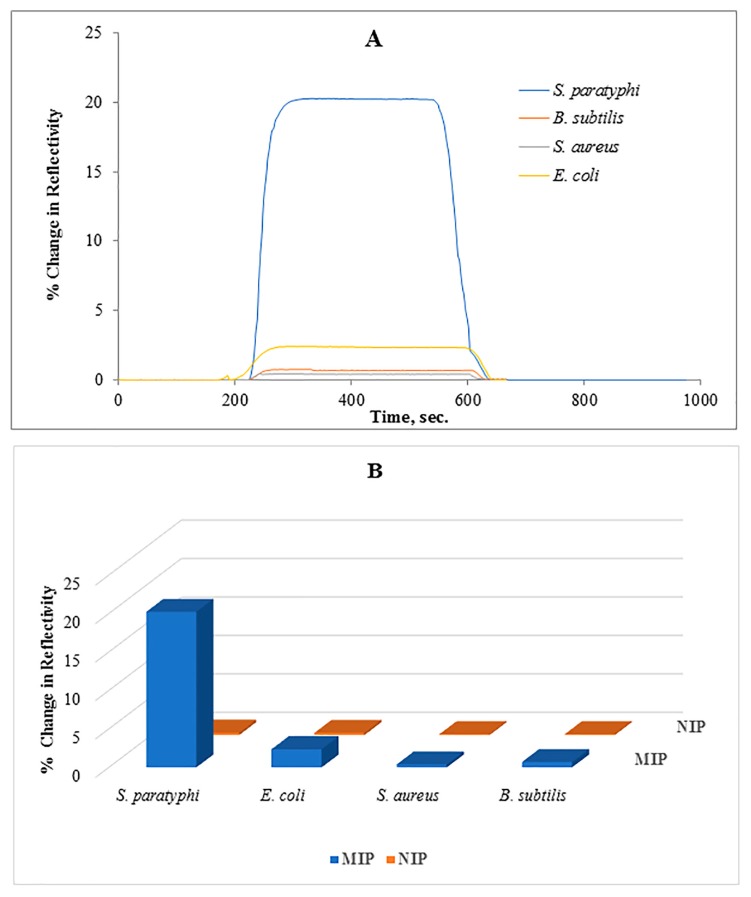
**(A**) Selectivity of microcontact-*S. paratyphi* imprinted SPR biosensor against competing bacterial strains; *Escherichia coli, Staphylococcus aureus, Bacillus subtilis*, (**B**) Imprinting efficiency of the microcontact-*S. paratyphi* imprinted SPR chips vs. non-imprinted SPR chips, experimental conditions; sample concentration: 1.5 × 10^7^ CFU/mL, flow rate: 200 µL/min, running buffer: PBS buffer, regeneration buffer: 10 mg/mL lysozyme solution, 50% ethyl alcohol.

The selectivity of generated SPR system was examined against *B. subtilis*, *S. aureus* and *E. coli* strains. Figure 5A shows the responses of sensor systems obtained from the application of all the tested bacterial strains to the microcontact-*S. paratyphi* imprinted SPR chips. As can be seen, ΔR values for competing bacterial strains were lower than that of *S. paratyphi*. Among these competing bacterial strains, the highest ΔR value was monitored after *E. coli* injection. *E. coli* belongs to the same family (Enterobacteriaceae) as *S. paratyphi* and has a similar size as *S. paratyphi.*

All tested bacterial strains other than *S. paratyphi* when introduced to the SPR chip caused low ΔR values in both MIP and NIP ones (Figure 5B). Higher responses against *S. paratyphi* indicated the unique characteristics of complementary cavities produced during the imprinting process. In comparison with non-imprinted ones, the responses against competitive bacterial strains were higher in imprinted sensors because of some similar features of the bacterial surfaces (Table 1). 

### 3.4. Real Sample and Reusability Studies

**Figure 6:**
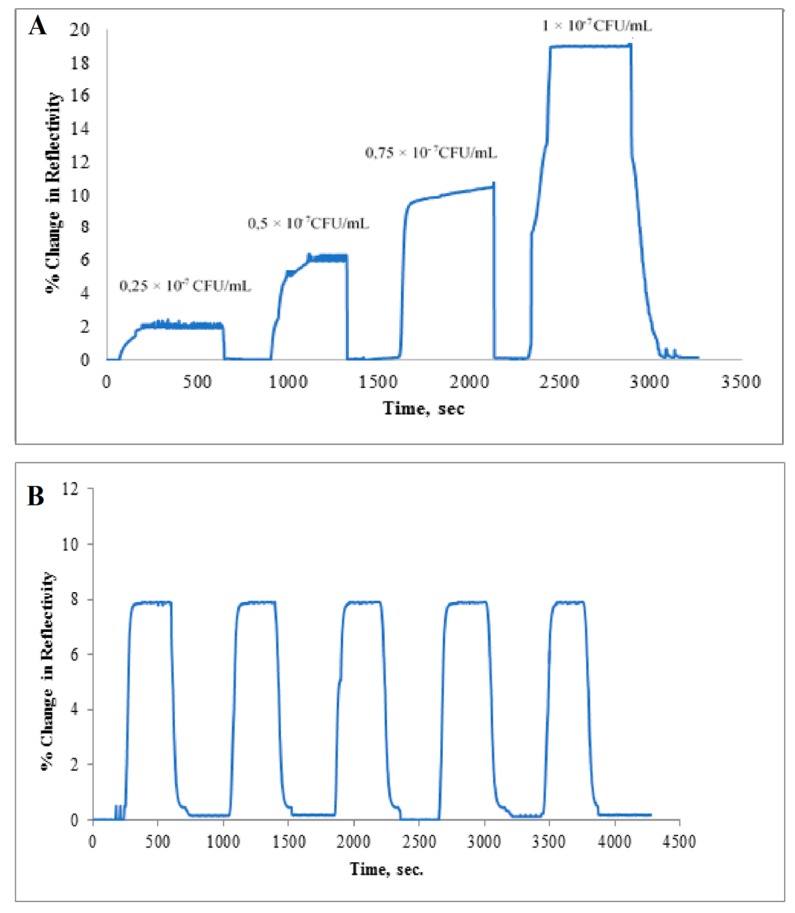
(**A**) Real-time responses of microcontact-*S. paratyphi* imprinted SPR chips against apple juice spiked with *S. paratyphi* at different concentrations in the range of 2.5 × 10^6^–10 × 10^6^ CFU/mL, (**B**) Reusability of microcontact-*S. paratyphi* imprinted SPR chips at the concentration of 7.5 × 10^6^ CFU/mL.

Real sample experiments were carried out with apple juice which provides ambient media for bacterial growth. The apple juice samples were spiked with *S. paratyphi* at a range of concentrations (2.5 × 10^6^–10 × 10^6^ CFU/mL). As seen in Figure 6A, the increase in concentration of *S. paratyphi* caused the increase in sensor response. 

The reusability of the microcontact*-S. paratyphi* imprinted sensors was examined with apple juice spiked with *S. paratyphi*. Equilibration–adsorption–regeneration cycles were repeated for five times at the concentration of 7.5 × 10^6^ CFU/mL (Figure 6B). 

Idil et al. preferred both *E. coli* spiked (1.0 × 10^2^–1.0 × 10^4^ CFU/mL) apple juice and river water samples in real sample experiments to indicate the applicability of the microcontact-*E. coli* imprinted capacitive sensor [4]. In another study, apple juice was selected as real sample and apple juice samples spiked with *E. coli* at different concentrations in the range of 0.5–4.0 McFarland (approx. 1.5 × 10^8^–12 × 10^8^ CFU/mL) were applied to the SPR system to confirm the developed sensor for *E. coli* detection [27]. Tokonami et al. used apple juice for real sample assays in order to verify if the generated system was able to detect target microorganisms at concentrations between 10^7^ to 10^9^ CFU/mL [35]. Son et al evaluated the feasibility of a miniature SPR sensor for detection of *Salmonella* enteritidis. Anti-*Salmonella* antibodies were attached on the SPR chip surface by using neutravidin. *Salmonella* was detected by SPR biosensor at concentrations down to 10^5^ CFU/mL [36]. Generation of *E. coli* imprints was successful using ready-to use materials as well as ab initio synthesized polyurethanes [37]. Dilutions of *E. coli* suspensions, down to a limit of detection of 1.4 × 10^7^ CFU/mL, were successfully measured using QCM.

It is from the data presented in esp. Figure 6B quite obvious that one can use the same sensor chip repeatedly, provided proper regeneration is carried out between the assays. As discussed earlier in this paper, ethanol in combination with lysozyme turned out to be efficient so that the sensor chip after proper treatment could be reconditioned in PBS buffer before a new assay cycle started.

## 4. Conclusions

There is a growing need for selective recognition of microorganisms in complex samples due to the recognition of the importance of detection of microbial contaminants. Rapid, reliable, specific and cost-effective devices providing real-time screening are required. In this respect, SPR biosensors address these requirements with the advantage of biosensing in cell recognition. Physical properties, such as the shape, size and charge of the cell surface can influence cell recognition. These may gain advantage from chemical characteristics including complementary recognition molecules in order to increase selectivity. These two properties have to be taken into account for the development of successful devices. MIPs appear as promising tools employing both physical and chemical properties suitable for the development of successful (stable and sensitive) systems. In this study, the microcontact imprinting technique provided a simple surface patterning procedure combined with a SPR system. The created biosensing system had a good performance and gave sensitive and selective responses to the target bacteria at a concentration range from (2.5 × 10^6^–15 × 10^6^ CFU/mL) with a CFU/mL detection limit and a linearity of *R*^2^ = 0.9925. In conclusion, the proposed sensor technology has the potential to serve as an excellent candidate for monitoring *S. paratyphi* in contaminated water or food supplies and clearly indicates that it is possible to develop rapid and appropriate control strategies.

The present paper clearly illustrates the potential of combining microcontact MIPs since one can achieve both shape and chemical matching binding zones. In the future one can expect microcontact MIPs with even higher selectivity to be used. This can be done by reaching an even better matching between the chemical structure in the MIP cavities and the surface of the target cells.

## Figures and Tables

**Table 1:** Selectivity coefficients of *S. paratyphi*-MIP and NIP SPR chips (ΔR: SPR response for the *S. paratyphi*-MIP and NIP SPR chips, *k*: selectivity coefficient for *S. paratyphi* versus competing bacterial strains, *k*′: relative selectivity coefficient for *S. paratyphi*-MIP SPR chips versus NIP SPR chips).

Bacterial Strains	SPR Response, ΔR	SPR Response, ΔR	Selectivity Coefficient, *k*	Selectivity Coefficient, *k*	Relative Selectivity Coefficient, *k*’
	Imprinted	Non-imprinted	Imprinted	Non-imprinted	
*S. paratyphi*	20.25	0.25	-	-	-
*S. aureus*	0.4	0.28	50.63	0.89	56.70
*E. coli*	0.7	0.35	28.93	0.71	40.50
*B. subtilis*	2.3	0.35	8.80	0.71	12.33
